# Microwave Absolute Distance Measurement Method with Ten-Micron-Level Accuracy and Meter-Level Range Based on Frequency Domain Interferometry

**DOI:** 10.3390/s23187898

**Published:** 2023-09-15

**Authors:** Longhuang Tang, Xing Jia, Heli Ma, Shenggang Liu, Yongchao Chen, Tianjiong Tao, Long Chen, Jian Wu, Chengjun Li, Xiang Wang, Jidong Weng

**Affiliations:** 1 Institute of Fluid Physics, China Academy of Engineering Physics, Mianyang 621900, China; jiaxing@caep.cn (X.J.); marcos12@126.com (H.M.); liushenggangpla@126.com (S.L.); ycchen16@fudan.edu.cn (Y.C.); zjuttj@163.com (T.T.); chenlongcaep@163.com (L.C.); ceuwj@zju.edu.cn (J.W.); lstrus@126.com (C.L.); xiangwang102@126.com (X.W.); 2 National Key Laboratory of Shock Wave and Detonation Physics, Institute of Fluid Physics, China Academy of Engineering Physics, Mianyang 621900, China

**Keywords:** absolute distance, broad-spectrum, frequency domain interferometry, microwave, precise ranging

## Abstract

A microwave absolute distance measurement method with ten-micron-level accuracy and meter-level range based on frequency domain interferometry is proposed and experimentally demonstrated for the first time. Theoretical analysis indicates that an interference phenomenon occurs instantaneously in the frequency domain when combining two homologous broad-spectrum microwave beams with different paths, and the absolute value of the distance difference between the two paths is only inversely proportional to the period of frequency domain interference fringes. The proof-of-principle experiments were performed to prove that the proposed method can achieve absolute distance measurement in the X-band with standard deviations of 15 μm, 17 μm, and 26 μm and within ranges of 1.69 m, 2.69 m, and 3.75 m. Additionally, a displacement resolution of 100 microns was realized. The multi-target recognition performance was also verified in principle. Furthermore, at the expense of a slight decrease in ranging accuracy, a fast distance measurement with the single measurement time of 20 μs was achieved by using a digitizer combined with a Fourier transform analyzer. Compared with the current microwave precision ranging technologies, the proposed method not only has the advantages of high precision, large range, and rapid measurement capability, but the required components are also easily obtainable commercial devices. The proposed method also has better complex engineering applicability, because the ten-micron-level ranging accuracy is achievable only by using a simple Fourier transform without any phase estimation algorithm, which greatly reduces the requirement for signal-to-noise ratio.

## 1. Introduction

Microwave precision ranging technology has attracted widespread interest in recent decades for various applications, such as target detection, precision machining control and assembly, liquid level monitoring, deformation or vibration monitoring, and internal flaw detection of non-metallic materials, due to its advantages over optical systems in terms of millimeter to sub-millimeter accuracy over the meter-scale range, anti-jamming, strong penetrability, and low path loss in environments with poor visibility or harsh conditions [1,2,3].

At present, different kinds of ranging methods, such as continuous wave (CW) interferometry [4], ultra-wideband (UWB) [5], and frequency modulated continuous wave (FMCW) [6], have been proposed to achieve millimeter to sub-millimeter accuracy in the microwave and millimeter wave bands. Among them, CW radar based on interferometry in the time domain allows the measurement of relative change in distance with micrometer-level accuracy, but the unambiguous range limited by the wavelength is only in the order of centimeters [7,8,9,10,11,12]. As for UWB radar, the technologies of two-stage sampling, increasing the number of base stations, high-fidelity crystal oscillators, and carrier down conversion demodulation have been used to obtain the absolute distance measurement ability of centimeter accuracy within the range of tens of meters, or millimeter accuracy within the range of several meters [13,14,15,16,17]. Compared with CW and UWB methods, FMCW radar is the most rapidly developing and widely used microwave ranging technique due to its balancing of range and accuracy. Recently, due to the progress of semiconductor technology, the operating bandwidth of FMCW radar has greatly increased from 1 GHz to 150 GHz [18,19,20,21,22,23]. The corresponding ranging accuracy analyzed with frequency-estimation algorithms, such as fast Fourier transform and Rife, has improved from the centimeter level to tens of microns within the range of a few meters. Additionally, a combined algorithm of frequency and phase estimation has been proposed and is widely used to improve ranging accuracy [24,25,26]. Due to the increase in microwave frequency, the accuracy of phase-based algorithms has been significantly improved. Therefore, with the improvement of the operating frequency from X-band to R-band, the ranging accuracy limit of the current FMCW radar has reached the micron or even sub-micron level. However, due to the requirement for precise phase information, this type of phase-based algorithm is only applicable to single-target ranging under conditions of a good signal-to-noise ratio. Additionally, the frequency sweeping time of microwave sources used in FMCW is mostly in the order of milliseconds, which makes it impossible to measure certain transient processes such as ultrafast-moving targets, high-frequency vibrations, and so on. Additionally, the high cost of large-bandwidth, high-linearity swept-frequency microwave sources and the high carrier frequency even up to the THz band also limit its practical applications to a certain extent.

In this study, a microwave absolute distance measurement method with ten-micron-level accuracy and meter-level range based on frequency domain interferometry is proposed and experimentally demonstrated in the X-band. To the best of our knowledge, this is the first time that the interference phenomenon in the frequency domain has been used to achieve precise ranging in the microwave band. The mechanism of microwave frequency domain interference (MFDI) ranging was quantitatively theoretically analyzed, and it indicated that the measured value of absolute distance is inversely proportional to the period of interference fringes in the frequency domain. Proof-of-principle experiments were performed using easily obtainable commercial devices to prove that the proposed method has the ability to realize absolute distance measurement in the X-band with a standard deviation of about 10–20 microns and a displacement resolution of better than 100 microns within the range of a few meters. The multi-target recognition performance was also verified in principle. Furthermore, a fast frequency domain interferogram recording method based on the digitizer combined with a Fourier transform analyzer was proposed to shorten the single measurement time to 20 microseconds at the expense of a slight decrease in measurement accuracy, which was nearly two orders of magnitude smaller than the frequency sweeping time of FMCW radar.

## 2. Theoretical Analysis

### 2.1. Ranging Principle of MDFI

**Figure 1:**
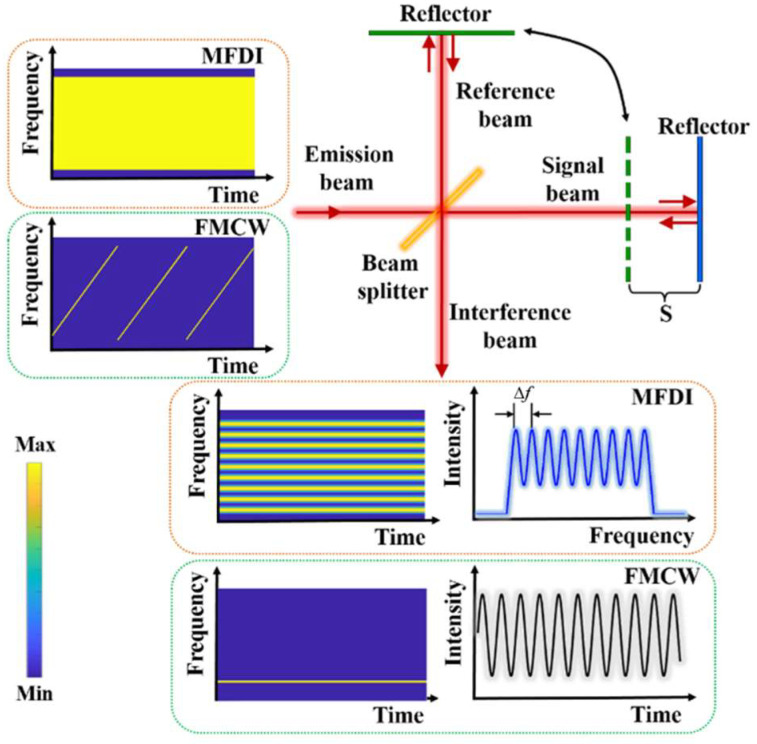
The principal comparison between MFDI ranging and FMCW radar.

Firstly, the basic principle of microwave frequency domain interference ranging is quantitatively explained based on the Michelson interferometric experimental structure. As shown in Figure 1, when a broad-spectrum microwave beam is emitted into the Michelson interferometer, the microwave field at any point *Q* on the interference beam is expressed as

(1) $$U_{Q}\left(t\right)=U_{1}\left(t-\tau_{1}\right)+U_{2}\left(t-\tau_{2}\right)$$

where the $U_{i}$ and $\tau_{i}$ (*i* = 1, 2) are the microwave field function and the transmission time on a path in the Michelson interferometer, respectively. Additionally, 1 and 2 represent the signal beam and reference beam, respectively.

The coherence function is introduced to analyze the interference characteristics of the superimposed microwave field [27]. The coherence function of point *Q* can be expressed as

(2) $$\Gamma_{Q}\left(\varphi \right)=\langle U_{Q}\left(t+\varphi \right)U_{Q}^{*}\left(t\right)\rangle$$

where φ is any time delay on the interference beam. < > and * are operations of ensemble average and complex conjugate, respectively. Equation (2) can describe the coherence between the microwave field transmitted after any time φ and its original field.

Then, substitute Equation (1) into Equation (2).

(3) $$\begin{array}{c} \Gamma_{Q}\left(\varphi \right)=\langle U_{1}\left(t-\tau_{1}+\varphi \right)U_{1}^{*}\left(t-\tau_{1}\right)\rangle +\langle U_{2}\left(t-\tau_{2}+\varphi \right)U_{2}^{*}\left(t-\tau_{2}\right)\rangle \\ +\langle U_{1}\left(t-\tau_{1}+\varphi \right)U_{2}^{*}\left(t-\tau_{2}\right)\rangle +\langle U_{2}\left(t-\tau_{2}+\varphi \right)U_{1}^{*}\left(t-\tau_{1}\right)\rangle \\ =\Gamma_{11}\left(\varphi \right)+\Gamma_{22}\left(\varphi \right)+\Gamma_{12}\left(\tau_{2}-\tau_{1}+\varphi \right)+\Gamma_{21}\left(\tau_{1}-\tau_{2}+\varphi \right) \end{array}$$

Here, $\Gamma_{ii}$ and $\Gamma_{ik}$ (*i, k* = 1, 2) represent the self-coherence function and the mutual coherence function, respectively. Additionally, let $\Delta \tau =\tau_{1}-\tau_{2}$, which is the transmission time difference between the signal beam and the reference beam.

Meanwhile, for generalized stochastic processes, the coherence function Γ(φ) and power spectral density function G(f) are the mutual Fourier transform pairs based on the Wiener–Khintchine theorem. Thus, the relation of $\Gamma \left(\varphi \right)\overset{F}{\underset{F^{-1}}{⇄}}G\left(f\right)$ is satisfied. Therefore, through Fourier transformation of Equation (3), the power spectral density function can be obtained as follows:

(4) $$\begin{array}{c} G_{Q}\left(f\right)=G_{11}\left(f\right)+G_{22}\left(f\right)+G_{12}\left(f'\right)\exp(-j2\pi f\Delta \tau )\\ +G_{21}\left(f'\right)\exp\left[-j2\pi f\left(-\Delta \tau \right)\right] \end{array}$$

where *f* is the microwave frequency. The $G_{ii}$ (*i* = 1, 2) is the power spectral density function, which represents the distribution of power in the frequency domain. Additionally, the $G_{ik}$ (*i*,*k* = 1, 2) is the cross-spectral density function, which represents the similarity degree of each frequency component *f* in the two stochastic processes of *i* and *k*.

Due to the relation of $G_{12}\left(f\right)=G_{21}^{*}\left(f\right)$ [23], the last term $G_{21}\left(f'\right)\exp\left[-j2\pi f\left(-\Delta \tau \right)\right]$ of Equation (4) can be expressed as ${\left[G_{12}\left(f'\right)\exp\left(-j2\pi f\Delta \tau \right)\right]}^{*}$. Based on the Euler formula Re[exp(jθ)]=cos(θ), the above equation can be further simplified as follows:

(5) GQ(f)=G11(f)+G22(f)+2Re[G12(f')]cos(2πΔτf)

The above equation represents the power spectral characteristics of an interference beam as a broad-spectrum microwave beam passes through the Michelson interference structure. Because the two superimposed microwave fields in the interference beam are homologous, their frequency components have a high similarity, which means that the value of $G_{12}\left(f'\right)$ is far greater than zero. Therefore, it can be deduced from Equation (5) that the power spectral density distribution of the interference beam is obviously not only equal to the sum of the spectral densities of two superimposed microwave fields, but also exhibits a cosine modulation effect in the frequency domain. This is the microwave frequency domain interference (MFDI) phenomenon.

According to Equation (5), for the frequency domain interference fringes, it can be seen that the period Δf as shown in Figure 1 is exactly equal to 1/Δτ, and the modulation depth is determined by the real part of cross-spectral density $G_{12}\left(f'\right)$.

Therefore, based on the characteristics of the frequency domain interference fringes described above, the transmission time difference Δτ can be directly obtained by performing Fourier transform on the frequency domain interferogram. Thus, the absolute value of the distance *S* between the signal arm and the reference arm of the Michelson interferometer could be calculated as follows:

(6) S=c×Δτ/2n

Here, *c* is the speed of light in a vacuum, and *n* is the refractive index of microwaves in the medium.

It can be seen from the above equation that the accuracy of the MFDI range is independent of carrier frequency *f*, and it only depends on the accuracy of Fourier transform when calculating Δτ, which can be improved by increasing the signal-to-noise ratio, number of sampling points of interference fringes, and operating frequency bandwidth. This makes it possible to select the appropriate carrier frequency for different applications without worrying about the impact of accuracy.

Furthermore, it is worth mentioning that the limitations of the MFDI ranging we have identified are analyzed as follows:

(a) The wider the operating bandwidth used by the proposed method, the higher the ranging accuracy. This requirement means that a broadband antenna needs to be used in the system. When the antenna bandwidth is several GHz, it has little effect on the measurement accuracy. However, when the antenna bandwidth is tens of GHz, due to the gradual deterioration of its directivity, a greater amount of environmental scattering noise will be introduced into the ranging measurement, which will affect the signal-to-noise ratio to a certain extent.
(b) According to Equation (5), the longer the measured distance is, the smaller the period of the frequency domain interference fringe, which makes the range limit depend on the frequency resolution of the frequency domain signal acquisition. In order to further improve the range, it is necessary to improve the resolution of spectrum acquisition, leading to an increased requirement for acquisition equipment.

### 2.2. The Principal Comparison

Based on the results of the above theoretical analysis, the principle of MFDI ranging is completely different from that of existing microwave ranging methods. Moreover, a brief comparison between MFDI ranging and FMCW radar, which is widely used at present, is shown in Figure 1 and described below.

(a) Different microwave sources are used. MFDI ranging uses a broad-spectrum microwave source that contains wide frequency components at any time. Additionally, FMCW radar uses a frequency-modulated microwave source whose frequency varies continuously with time. Compared to the FMCW radar, which needs a high-linearity frequency ramp in the wide bandwidth, MFDI can greatly reduce the requirements of high-quality microwave sources in the current microwave precision ranging system.
(b) The interference phenomena occur in different domains. For MFDI ranging, the interference fringes appear in the frequency domain. Because all the frequency components exist at any given time, the interference fringe is complete at any time. It therefore has the potential to realize ultrafast distance measurement. As for FMCW radar, the power spectrum of the interference beam has only one intermediate-frequency signal component in the frequency domain, so its interference fringes only appear in the time domain. Therefore, the time needed to achieve a complete interference fringe acquisition depends on the frequency sweep time of its microwave source, which is usually in the order of milliseconds.
(c) The factors that determine the measurement accuracy are different. For MDFI ranging, the accuracy only depends on the signal-to-noise ratio, the number of sampling points, and the operation bandwidth, while that of FMCW radar is not only related to the same factors above, but also to the frequency ramp non-linearity and phase noise of the microwave source [28].
(d) The factors that determine the measurement sensitivity are the same. Whether it uses MDFI ranging or FMCW radar, the sensitivity of the system depends on the minimum signal power that the low-noise amplifier can effectively amplify in the receiving link, which is often positively correlated with the noise figure and noise temperature of the amplifier.

## 3. Experimental Setup

**Figure 2:**
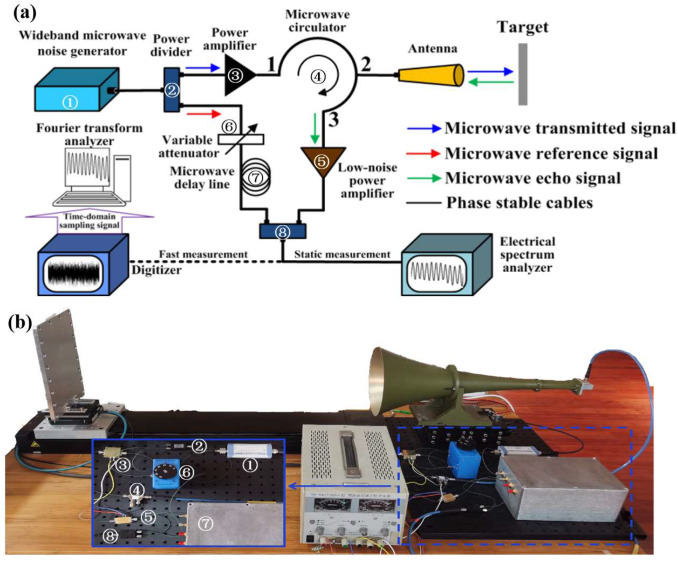
(**a**) The experimental setup of MFDI ranging. (**b**) The photo of MFDI ranging system.

A proof-of-concept experimental setup of the microwave absolute distance measurement method based on frequency domain interference was built to verify the performance in terms of accuracy and range. It is shown in Figure 2. The same number of the device in the schematic diagram and the photo is convenient for identification. A wideband microwave noise generator (REBES: NW346KA, from Suzhou, China) with a center frequency of 10 GHz and a bandwidth of 20 GHz was used as the broad-spectrum microwave source. Through a power divider (LIAR: LPD2S-1-18, from Suzhou, China, isolation of 16 dB, insertion loss of 1.2 dB), the microwave beam was divided into two parts, which were named the transmitted signal and the reference signal, respectively. The power of the transmitted signal was amplified to about 10 dbm using a power amplifier (TALENT: TLPA50K20G-32-22, from Suzhou, China, gain of 32 dB). A horn antenna (HENGDA: HD-23.825CHAX, from Xi’an, China, gain of 18 dB, VSWR of 1.5) connected to a microwave circulator (REBES: D4C8012, from Suzhou, China, isolation of 20 dB, insertion loss of 0.5 dB) was used to emit and receive the transmitted signal and its echo simultaneously. After the port 3 of the circulator, the power of the echo signal was improved by a low-noise power amplifier (TALENT: TLLA0.1G26.5G-18-60, from Suzhou, China, gain of 18 dB). Then, the echo and reference beams were coupled using a power combiner (LIAR: LPD2S-1-18, from Suzhou, China, isolation of 16 dB, insertion loss of 1.2 dB) to form the frequency domain interferogram, which was recorded with an electrical spectrum analyzer (ESA, CEYEAR: 4051-S, from Qingdao, China,) or a digitizer combined with a Fourier transform analyzer. To obtain a high-quality frequency domain interferogram, the balance between the power of the echo and reference signals was achieved by adjusting the variable attenuator (REBES: RBS-69-26.5-7, from Suzhou, China, attenuation region of 0–69 dB in 1 dB steps, insertion loss of 2 dB). All devices were connected with a cable (TALENT: T1-SMAMSMAM-L, from Suzhou, China, insertion loss of 3 dB). Meanwhile, a microwave delay line was used to pre-eliminate the initial phase difference between transmitted and reference signals that was introduced by the system. This ensured that the ranging result was an absolute distance value from the antenna port to the target. The target used was a plane-parallel metal plate with a size of 30 cm by 20 cm, which was mounted on a motorized translation stage with an accuracy better than 1 μm. It is worth mentioning that the spectrum range of the stable frequency domain interferogram in our experiment was limited by the bandwidth of the microwave circulator, whose operated frequency range was from 8 GHz to 11 GHz. Furthermore, a preliminary analysis of the link budget was made. Since the absorption coefficient of the air to microwave in the X-band is about 0.005 dB/km, the influence of atmospheric absorption can be ignored in the meter-scale range. In this link budget, the loss mainly comprised the insertion loss of the device. The microwave power after the power amplifier was 10 dBm. Considering that the insertion loss of the microwave circulator and cable and the VSWR of the antenna were 3.5 dB and 1.5, respectively, the transmit power $P_{t}$ of the antenna was 5.53 dBm. The RCS σ of the used plane-parallel metal plate target in the X-band was calculated to be 50.27 m^2^. Considering that the antenna gain *G* was 18 dB, the echo power $P_{r}$ received by the antenna could be calculated using the radar equation as follows:

(7) $$P_{r}=P_{t}G^{2}\lambda^{2}\sigma /{\left(4\pi \right)}^{3}R^{4}$$

where λ and R are the microwave wavelength and detected distance, respectively.

In our experiment, the maximum distance R was about 3.7 m. The minimum echo power $P_{r}$ was calculated to be −38.51 dBm. Additionally, the insertion loss of the cable was 3 dB. Therefore, the echo signal power before entering the low-noise power amplifier was about −41.51 dBm, which was much higher than the minimum signal power of −68.57 dBm required by the low-noise power amplifier. Therefore, this system can be expected to obtain good frequency domain interference fringes.

## 4. Results and Discussion

**Figure 3:**
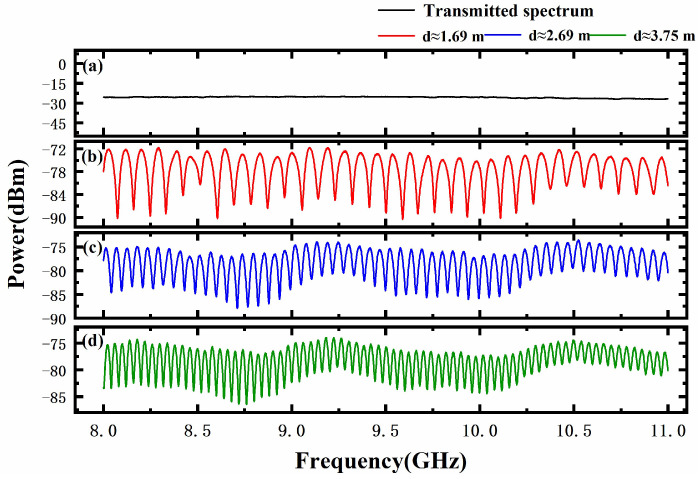
(**a**) The transmitted microwave spectrum. (**b**–**d**) The measured microwave frequency domain interferogram at different distances.

Firstly, the transmitted microwave spectrum and the microwave frequency domain interferogram were measured with the electrical spectrum analyzer with resolution bandwidth of 10 MHz, video bandwidth of 10 kHz, and 20,000 sample points. As shown in Figure 3a, the transmitted signal had a relatively flat power curve in the range from 8 GHz to 11 GHz, and the difference between the maximum and minimum power was only 2.29 dB. The frequency domain interferogram at the absolute distances of about 1.69 m, 2.69 m, and 3.75 m is recorded, and the distances are shown in Figure 3b–d, respectively. It is clear that all the frequency domain interference fringes have a modulation depth of more than 10 dB with a good signal-to-noise ratio. Additionally, the longer the measurement distance, the smaller the interference fringe period, which is consistent with our previous theoretical analysis. Moreover, the same envelope modulation phenomenon was found in three interferograms, which was caused by the frequency domain interference occurring between the reference signal and the undesired return signal from the antenna feed connector with a return loss of 13 dB. However, since their path distances differ by only a few tens of centimeters, the slowly varying envelope modulation could not be avoided in this experiment, but it can be removed using Fourier transform without affecting the measuring range and accuracy.

**Figure 4:**
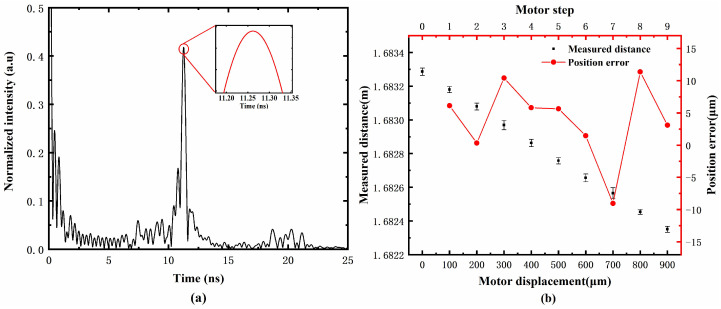
(**a**) The Fourier transform analytical spectrum of the measured microwave frequency domain interferogram. (**b**) The distance measurement results and error as a function of motor displacement in 100 μm steps.

According to Equation (6), the measured absolute distance could be calculated by using the Fourier transformed result of the microwave frequency domain interferogram shown in Figure 3. The typical Fourier transform analytical spectrum is shown in Figure 4a. It can be seen that the maximum peak is located at 11.262 ns, corresponding to the measured distance of 1.68928 m. Since no microwave-absorbing material was used in this experiment to suppress multipath reflections, three low side lobes appear near the main peak in the Fourier transform spectrum. Additionally, some small peaks appearing near 20 ns were frequency-multiplied signals caused by the multiple reflections between the target and the antenna. Furthermore, the displacement resolution was characterized by measuring the absolute distance of the target controlled by the motorized translation stage with a step size of 100 μm. For each target position, repeated distance measurements were taken 10 times, and the results are shown as the error bars in Figure 4b. Additionally, the average of every ten measurements was calculated as the measurement result. Compared with the motor displacement, the measurement errors were less than ±12 μm. Based on the above experimental results, the proposed MFDI ranging method had been proven to have the ability to measure small displacements of tens of micrometers in a meter-scale range.

**Figure 5:**
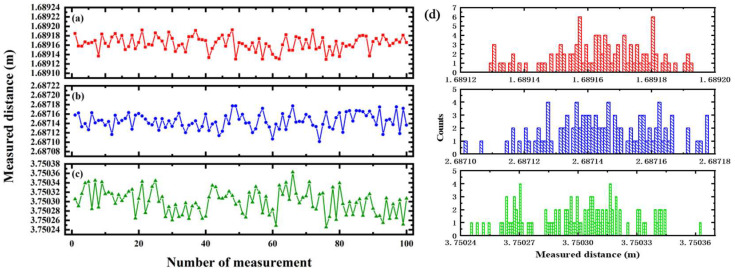
The measured distance stability of a hundred measurements at about (**a**) 1.69 m, (**b**) 2.69 m, and (**c**) 3.75 m. (**d**) The distribution histogram of measured values at different distances.

In order to better evaluate the measurement stability at different ranges, the absolute distance measurement was repeated 100 times for statistical calculation at each position. The repeated measurement results at three distances are shown in Figure 5. The standard deviation at the measured distances of 1.69 m, 2.69 m, and 3.75 m were calculated to be 15 μm, 17 μm, and 26 μm, respectively. Because the standard deviation *σ* is directly related to the measurement uncertainty $u_{a}$, we take the 95% confidence interval as an example, $u_{a}=2\sigma /\sqrt{N}$, where *N* is the number of measurements. The distance values were expressed as the measured mean ± the uncertainty, which were 1.689163 m ± 3 μm, 2.687145 m ± 3 μm, and 3.750299 m ± 5 μm (95% confidence interval), respectively. It is clear that, although the standard deviation of the measured distance increased slowly with the increase in measuring range, the measurement stability remains at about 20 μm. The distribution characteristics of the measured values at three distances were statistically analyzed, and the results are displayed in the histogram in Figure 5d, which shows that the measured distance values basically obey the normal distribution. It can be inferred that the measurement stability of a long-range target will be further improved by increasing the signal-to-noise ratio of frequency domain interference signals via the transmitted microwave power enhancement.

**Figure 6:**
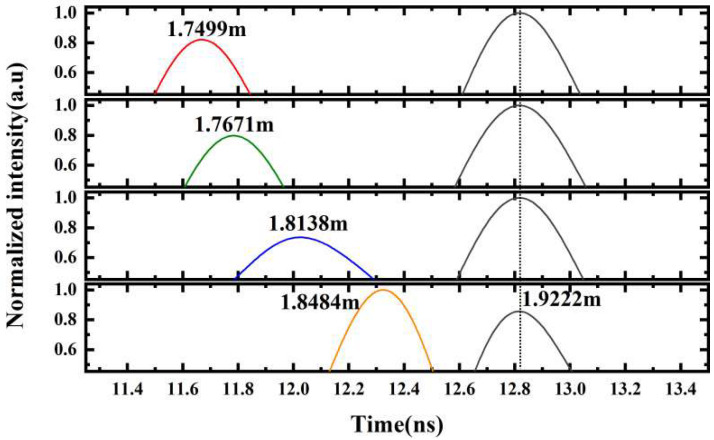
The multi-target recognition in the Fourier transform analytical spectra.

Furthermore, the multi-target recognition based on the proposed method was experimentally demonstrated. In this experiment, the target in Figure 2 was replaced by two parallel metal plates with a certain initial spacing, both of which were covered by half of the transmitted microwave beam. By moving one of the plates, the microwave frequency domain interferogram of two targets with different spacing was measured. The Fourier transform analytical spectra are shown in Figure 6. It can be seen that one of the targets was fixed at about 1.92 m, while the other was gradually approaching. When the spacing varied from 17.23 cm to 7.38 cm, the two targets were clearly recognized in the spectra. According to the Fourier transform theory, only two distance signals with a time delay greater than 1/B (microwave frequency range B was 3 GHz in this experiment) can be completely distinguished in the spectrum. Therefore, the theoretical limit of the resolvable spacing between the two targets in this experiment was 5 cm, which is basically consistent with the experimental results above. It can be inferred that the spacing recognition capability of multiple targets based on the MFDI ranging could be further improved to the sub-millimeter level by easily increasing the frequency operation bandwidth to the order of ten GHz.

**Figure 7:**
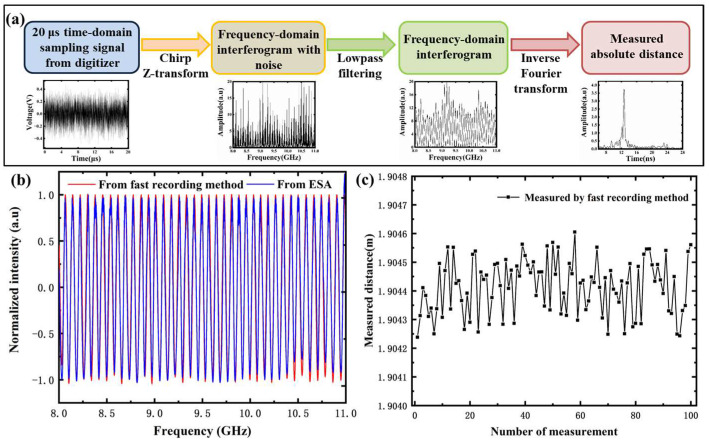
(**a**) The process of the fast interferogram recording method. (**b**) The comparison of interference fringes recorded using the fast recording method and ESA. (**c**) The measured distance stability of the fast recording method.

According to the above theoretical analysis, the establishment of frequency domain interference fringes in MFDI ranging is instantaneous, which gives it the potential of ultrafast process measurement. This is especially useful in the field of military industrial applications, such as in the measurement of the combustion process of explosives, the propagation process of shock waves in non-metallic materials, and the displacement history of the flyer during the explosion damage process. Because these durations are only milliseconds in length, it requires a fast microwave ranging capability with a microsecond response. However, limited by the spectrum acquisition time of the electrical spectrum analyzer, the single measurement time of MDFI ranging was about one second. In order to improve the fast measurement ability of MFDI ranging, a fast interferogram recording method based on the digitizer combined with a Fourier transform analyzer (a self-compiled Fourier transform processing program operated via a computer) was proposed and demonstrated experimentally. The process is shown in Figure 7a. The frequency domain interference signal was first sampled directly using the digitizer with a sampling rate of 50 GS/s, and it looked similar to the noise signal in the time domain. Then, the frequency domain interferogram was obtained by performing the chirp Z-transform and low-pass filtering on the time domain sampling data in 20 ms. As shown in Figure 7b, the normalized comparison of interference fringes obtained at the same distance using the fast recoding method and ESA showed good agreement. Moreover, the measurement stability of the above method was evaluated via repeated ranging of 100 times with the single sampling time of 20 microseconds, and the measured values are shown in Figure 7c. The distance value was 1.904414 m ± 18 μm (95% confidence interval). The average value of the repeated measurements was 1.90441 m, which was consistent with the ESA measurement result of 1.90436 m. The standard deviation was calculated to be 96 μm. It is clear that the above experimental results indicate that the proposed fast measurement method could improve the measurement repetition rate from 1 Hz to 50 kHz at the expense of a slight decrease in ranging accuracy.

In order to provide a more in-depth comparison and evaluation of the proposed method against existing techniques, the different microwave precision ranging methods were compared in terms of carrier center frequency, bandwidth, algorithm, ranging distance, displacement, measurement error, single measurement time, and unambiguous distance, as shown in Table 1. Since the performance of some methods is directly related to the carrier frequency, in order to make a more reasonable comparison, the works in the X-band or adjacent bands were selected as much as possible. It can be seen that the MFDI ranging method could realize a promising balance between ten-micron-level accuracy, meter-level range, and microsecond-level measurement rate.

## 5. Conclusions

In conclusion, a simply structured and easy-to-operate method of precise microwave ranging based on frequency domain interferometry has been demonstrated for the first time in this study. The theoretical analysis indicates that the absolute distance value is only inversely proportional to the period of interference fringes in the frequency domain and independent of the operating frequency. The results of the proof-of-principle experiment prove that the proposed method has the ability to realize absolute distance measurement with standard deviations of 16 μm, 17 μm, and 26 μm in the range of 1.69 m, 2.69 m, and 3.75 m, respectively. Moreover, a displacement resolution of 100 μm was achieved at the distance of 1.69 m, corresponding to measurement errors less than ±12 μm. The multi-target recognition performance was also verified in principle. Moreover, in order to overcome the longer recording time of the interferogram using ESA, the fast measurement method was proposed and demonstrated to shorten the single measurement time from 1 s to 20 ms, with a distance measurement standard deviation of less than 100 microns. Since the ten-micron-level ranging accuracy can be achieved only by using the simple discrete Fourier transform without any phase estimation algorithm, this method has a low requirement in terms of the signal-to-noise ratio of the interferogram, which is suitable for complex engineering environments. Thus, it is expected that the proposed method can play an important role in broader microwave precision ranging applications.

**Figure 8:**
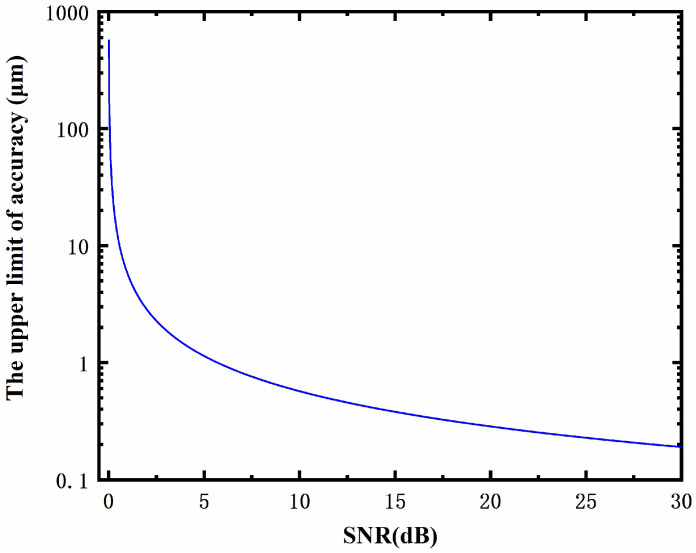
The theoretical upper limit of distance accuracy of MFDI.

In future research, the influence of operating bandwidth and signal-to-noise ratio on ranging accuracy and resolution will be quantitatively analyzed in theory and experimentally verified to provide guidance for the optimization of system parameters. Specifically, according to Equation (6), the absolute distance measurement realized via the proposed method depends only on the calculation of Δτ. Therefore, the accuracy and resolution of distance measurement are determined by the calculation accuracy of Δτ, which is related to the hardware parameters of the experimental system and the signal processing algorithm. In this manuscript, the conventional Fourier transform algorithm was used to solve Δτ. Its resolution and limitation of accuracy are equal to fs/N (where fs is the frequency spectrum sampling rate and *N* is the number of sampling points) and var(∆τ)×c/2 (where the Cramér–Rao lower bound $var\left(∆\tau \right)=12/[4\pi^{2}\eta N\;(N^{2}-1)]$, η is the signal-to–noise ratio), respectively. In our experiment, the number of sampling points *N* was 20,000, and SNR η was usually 0–30 db. Therefore, the theoretical upper limit of distance accuracy at different SNRs was calculated using var(∆τ)×c/2, and this is shown in Figure 8. It can be seen that the measurement accuracy of the current system has the potential to be further improved. Meanwhile, it is worth mentioning that the fs/N is also equal to the operating frequency bandwidth. Therefore, with the further increase in current experimental hardware parameters such as signal-to–noise ratio, number of sampling points, and operating frequency bandwidth, the accuracy and resolution of distance measurement could be improved.

On the other hand, solving Δτ is essentially a frequency estimation problem of a sinusoidal signal with finite length. Therefore, a lot of signal processing algorithms could also be considered to improve ranging accuracy and resolution. For instance, without considering multi-target resolution, some frequency estimation algorithms, such as maximum likelihood estimation, MUSIC, and Rife, or initial phase estimation algorithms can be used to process frequency domain interference signals. However, the effect on improving ranging accuracy and resolution needs to be further studied.

Additionally, ranging systems operating in different microwave bands will also be developed to further meet the different needs in practical applications. The development of microwave and terahertz wave electronic technology has resulted in mature commercial products for most of the devices needed in the proposed method. However, there are currently no ready-made products for broadband microwave noise sources above 40 GHz. Hence, one of the crucial directions for future research is to generate broadband microwave/terahertz wave radiation with arbitrary frequency band clipping using microwave photonics methods. This will facilitate the expansion of MDFI ranging systems in various frequency bands.

## Figures and Tables

**Table 1:** Performance comparison of different microwave precision ranging methods.

Center Frequency and Bandwidth (GHz)	Method and Algorithm	Measured Distance and Displacement	Measurement Error	Single Measurement Time and Unambiguity Distance	Ref.
24 Single tone	CW Phase-estimation	1 m 10 μm	0.5 μm	Not mentioned 12.5 mm	[9]
78 Two tones	AMCW ^a^ Phase-estimation	0.4 m 2 mm	8 μm	1.6 μs 15 cm	[12]
80 2	PMCW ^b^ Phase-estimation	0.6 m 1 mm	7 μm (400 times on average)	2 μs >1 km	[29]
9.5 1	FMCW Frequency-estimation	1–6 m 2 mm	100 μm	100 ms >1 km	[19]
24 1	FMCW Frequency- and phase-estimation	0.1 m 1 mm	35 μm (60 times on average)	500 μs >1 km	[25]
24.3 1	FMCW Frequency- and phase-estimation	1.4 m 1 mm	5 μm (500 times on average)	1 ms >1 km	[26]
9.5 3	MDFI Frequency-estimation	1.6 m 100 μm	12 μm (10 times on average)	20 μs >1 km	This work

^a^ AMCW: amplitude-modulated continuous wave radar. ^b^ PMCW: phase-modulated continuous wave radar. Since the related work of AMCW and PMCW ranging was not realized in the X-band, the recent works of W-band were selected for comparison. Meanwhile, it is worth mentioning that, for the ranging method based on phase estimation algorithm, the higher the carrier frequency, the higher the accuracy.

## Data Availability

Not applicable.
